# Comparing single-shot damage thresholds of boron carbide and silicon at the European XFEL

**DOI:** 10.1107/S1600577524007318

**Published:** 2024-08-25

**Authors:** Marziyeh Tavakkoly, Jaromir Chalupsky, Vera Hajkova, Wolfgang Hillert, Simon Jelinek, Libor Juha, Mikako Makita, Tommaso Mazza, Michael Meyer, Jacobo Montano, Harald Sinn, Vojtech Vozda, Maurizio Vannoni

**Affiliations:** a European XFEL Holzkoppel 4 22869Schenefeld Germany; b Institute of Experimental Physics Universitat Hamburg Luruper Chaussee 149 22761Hamburg Germany; c FZU – Institute of Physics, Czech Academy of Sciences, Na Slovance 2, 18221Prague 8, Czechia; d Deutsches Elektronen-Synchrotron Notkestrasse 85 22607Hamburg Germany; e Institute of Plasma Physics, Czech Academy of Sciences, Za Slovankou 3, 18200Prague 8, Czechia; f Faculty of Mathematics and Physics Charles University Ke Karlovu 3 12116Prague 2 Czechia; Paul Scherrer Institut, Switzerland

**Keywords:** damage threshold, single-shot damage threshold, B_4_C coating, X-ray mirrors, XFEL

## Abstract

Xray free-electron lasers (XFELs) enable experiments that would have been impractical or impossible at conventional X-ray laser facilities. Indeed, more XFEL facilities are being built and planned, with their aim to deliver larger pulse energies and higher peak brilliance. While seeking to increase the pulse power, it is quintessential to consider the maximum pulse fluence that a grazing-incidence FEL mirror can withstand. To address this issue, several studies were conducted on grazing-incidence damage by soft X-ray FEL pulses at the European XFEL facility. Boron carbide (B_4_C) coatings on polished silicon substrate were investigated using 1 keV photon energy, similar to the X-ray mirrors currently installed at the soft X-ray beamlines (SASE3). The purpose of this study is to compare the damage threshold of B_4_C and Si to determine the advantages, tolerance and limits of using B_4_C coatings.

## Abstract 2

The damage threshold fluences of silicon (Si) and boron carbide (B_4_C)-coated Si at 1 keV and 9 mrad grazing incident angle are compared. Results show that B_4_C has a damage threshold approximately twice that of Si, highlighting the advantages of using B_4_C coatings for enhanced durability.

## 1. Introduction

X-ray free-electron laser (XFEL) facilities have the capability to produce high-brightness X-rays, enabling novel experiments to be performed (Tschentscher *et al.*, 2017 ▸). The optical components of XFEL beamlines, such as mirrors and crystals, are constantly exposed to extremely intense photon beams concentrated in an ultrashort pulse duration (fs) – a situation that could easily cause irreversible damage to their surfaces, leading to compromised performance or to an unusable state altogether. A critical question therefore needs to be asked – what is the maximum pulse fluence that a grazing-incidence FEL mirror can withstand under continuous photon impingement with MHz repetition rate at an accelerator-based light source such as the European XFEL (EuXFEL).

To address this question, we conducted grazing-incidence damage experiments for uncoated silicon substrates and B_4_C coated substrates, using the EuXFEL facility and in particular its soft X-ray beamline at 1 keV photon energy. We chose silicon as the substrate and B_4_C as the surface coating as this combination is most commonly used at soft X-ray FELs beamlines (Mazza *et al.*, 2012 ▸; Tavakkoly *et al.*, 2022 ▸). A higher damage threshold fluence for the B_4_C coating is expected due to its good thermal properties, high melting point, low density and high reflectivity.

Previous studies have explored the damage threshold of similar materials under various conditions. For instance, experiments with uncoated silicon under normal incidence at 10 keV reported a damage threshold fluence, *F*_th_, of 0.78 µJ µm^−2^ (Koyama *et al.*, 2015 ▸). At a grazing angle of 22 mrad and 0.9 keV, silicon exhibited an *F*_th_ of 0.014 µJ µm^−2^ (Krzywinski *et al.*, 2018 ▸). Another study at 5.5 keV and an incident grazing angle of 11.4 mrad found a threshold fluence of 0.052 µJ µm^−2^ (Koyama *et al.*, 2016 ▸). For bulk B_4_C at normal incidence and 0.83 keV photon energy, the threshold fluence was 0.027 µJ µm^−2^ (Hau-Riege *et al.*, 2010 ▸). Moreover, a 50 nm B_4_C coating on a silicon substrate at 7 keV and an incident grazing angle of 4 mrad exhibited a damage threshold of 24 µJ µm^−2^ (Aquila *et al.*, 2015 ▸).

In this paper, we investigate the damage threshold of uncoated silicon and B_4_C coating for a grazing-incident angle of 9 mrad. The results of these experiments will provide critical insights into the durability and performance limits of these materials under extreme conditions, which is essential for optimizing the design and operation of XFEL beamlines.

## 2. Experimental setup

The experiment was conducted at the Small Quantum Systems (SQS) instrument at the EuXFEL (Mazza *et al.*, 2012 ▸). The facility delivered X-ray pulses with an average pulse energy of approximately 4.7 mJ and pulse duration of 25 fs at a photon energy of 1 keV. The samples analyzed in this study were a polished silicon wafer without any coating and a similar silicon substrate coated with B_4_C. The samples’ dimensions were 29.8 mm in length, 19.8 mm in width and 1830–1870 µm in thickness, with a 50 nm-thick B_4_C layer.

Samples were irradiated at a grazing angle of 9 mrad, which is typically used for B_4_C coated mirrors at soft X-ray beamlines. This angle is below the critical angle of 32 mrad at 1 keV (https://henke.lbl.gov/tmp/xray1041.dat).

**Figure 1:**
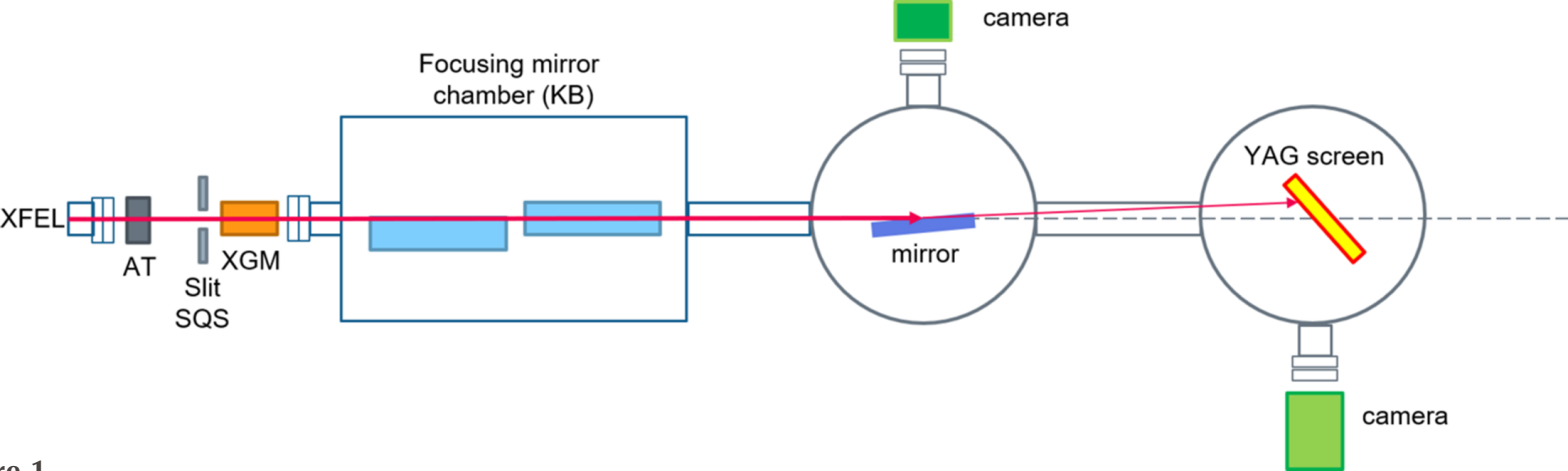
Schematic of the experimental setup. AT: attenuator; XGM: X-ray gas monitor.

The experimental setup is shown in Fig. 1 ▸. A gas attenuator was used to adjust the pulse energy and therefore the fluence. The maximum deliverable pulse energy to the samples was 1.8 mJ. An X-ray gas monitor (XGM) detector recorded the incident pulse energy for each damage measurement (Mazza *et al.*, 2012 ▸). The shot-to-shot pulse energy fluctuation was measured to be 5%.

A pair of Kirkpatrick–Baez (KB) optics was utilized to focus the beam, resulting in a spot size with an effective area of 1590 µm^2^. In this experimental setup, two cross chambers were used, one for the sample and the other, at 700 mm downstream from the first chamber, for the YAG screen. The YAG screen in the second chamber was used to track the direct beam and its reflection.

**Figure 2:**
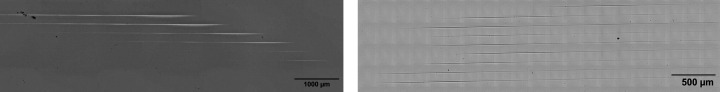
Representative single-shot damage craters at 9 mrad grazing-incidence angle. (Left) Silicon. (Right) B_4_C.

At each different attenuation settings, 43 damage measurements were taken at a grazing angle of 9 mrad. Fig. 2 ▸ illustrates a representative set of damaged craters.

## 3. Analysis and results

In order to obtain the threshold fluence, the first step is to determine the corresponding energy for damage threshold (Dastjani Farahani *et al.*, 2011 ▸; Koyama *et al.*, 2015 ▸). We measured the areas of the damaged spots in grazing-incidence irradiation for this purpose.

**Figure 3:**
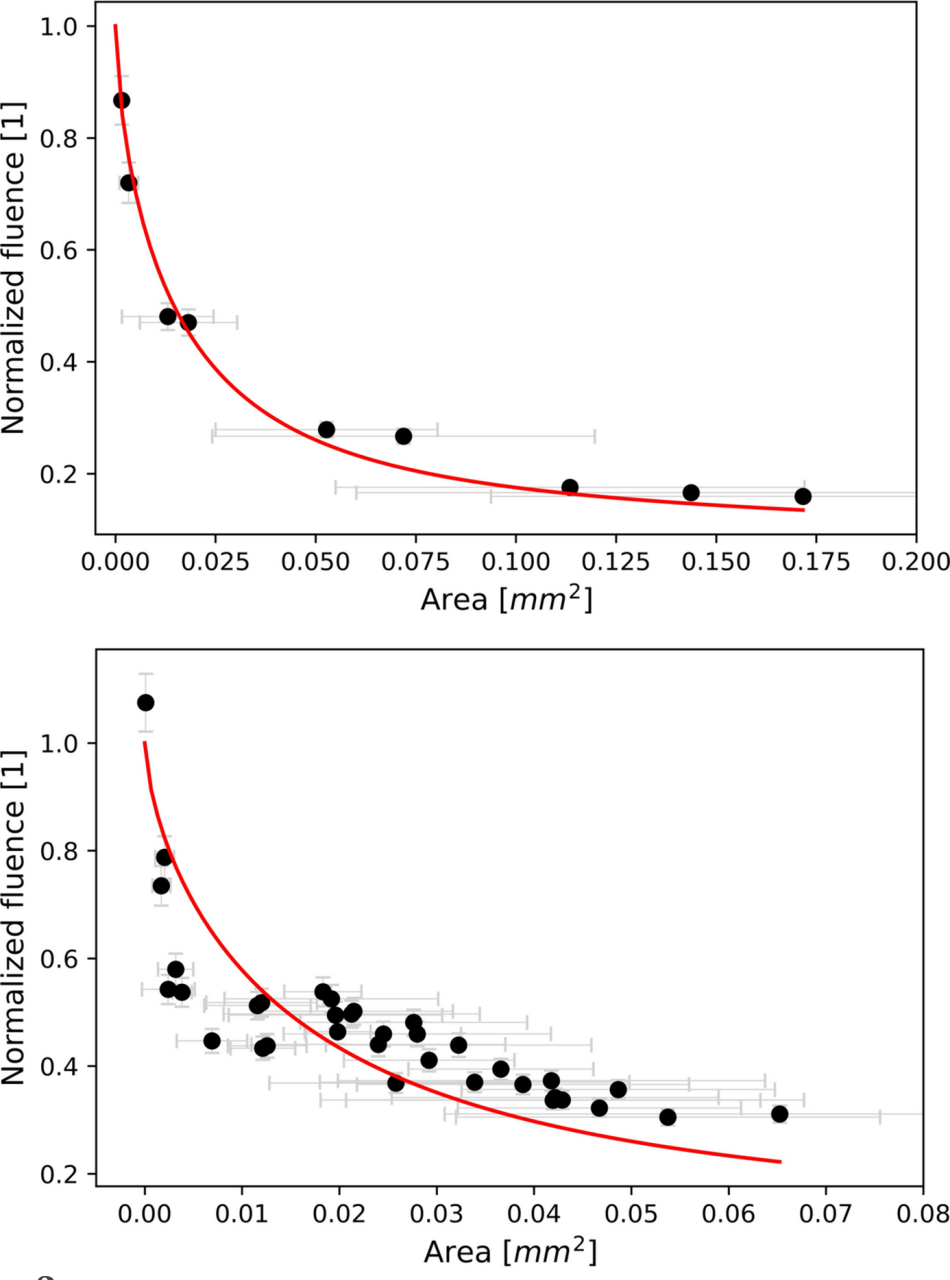
Normalized fluence versus damaged area at 1 keV. Each damaged area was measured for every pulse energy, with the red curve representing the beam profile. (Top) Silicon with threshold energy of 261 µJ. (Bottom) B_4_C with threshold energy of 549 µJ.

When the beam has a perfect Gaussian shape, we can determine the threshold energy by fitting the area of the damaged spot to the logarithm of the pulse energy. The maximum pulse energy at which damage does not occur is determined by the point of intersection in the linear fit (Liu, 1982 ▸; Aquila *et al.*, 2013 ▸). For non-Gaussian beams, the threshold energy is determined by minimizing the mean-square distance between experimental data points and a function of the beam profile in a plot of normalized fluence against the beam area. The beam function is determined using the procedure described by Chalupský *et al.* (2009 ▸, 2010 ▸),
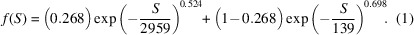
Here, *S* represents the area of the ablation contour, and *f*(*S*) denotes the normalized fluence. We measured the damaged area for each pulse energy. Fig. 3 ▸ presents a plot of normalized fluence versus measured damaged area, with the red curve depicting the beam profile.

As a result of our studies the determined threshold energies for silicon and B_4_C are 261 µJ and 549 µJ, respectively.

Finally, to retrieve the threshold fluence *F*_th_, one needs to determine the beam effective area. According to Chalupský *et al.* (2010 ▸), the effective area *A*_eff_ is determined by a method of ablation imprints and is defined by a relation between fluence and pulse energy, *F* = *E*_pulse_/*A*_eff_. It was found to be 1590 µm^2^. As a result, the threshold fluence (*F*_th_ = *E*_th_/*A*_eff_) is 0.16 µJ µm^−2^ for Si and 0.34 µJ µm^−2^ for B_4_C.

## 4. Conclusion

Single-shot damage measurements were conducted on Si and B_4_C-coated Si to determine the damage threshold fluences. At 1 keV with a 9 mrad grazing angle, below the critical angle, the damage threshold fluence was determined to be 0.16 µJ µm^−2^ for Si and 0.34 µJ µm^−2^ for B_4_C. As expected, B_4_C has a damage threshold approximately twice that of Si.

## Data Availability

The authors confirm that the data supporting the findings of this study are available within the article and its supplementary materials.
